# Lessons of screening two million newborns for congenital adrenal hyperplasia: 10-year experience of the Minas Gerais Public Health Program

**DOI:** 10.1016/j.jped.2024.10.012

**Published:** 2025-03-15

**Authors:** Cristina Botelho Barra, Gabrielly Souza Sena, Helena Pereira Oliveira, Ana Luiza Ataíde Carneiro de Paula Gonzaga, Raquel Ferreira Araújo, Thais Ramos Villela, Rafael Machado Mantovani, José Nélio Januário, Ivani Novato Silva

**Affiliations:** a Universidade Federal de Minas Gerais, Hospital das Clínicas, Serviço de Endocrinologia Pediátrica, Belo Horizonte, MG, Brazil; b Universidade Federal de Minas Gerais, Belo Horizonte, MG, Brazil

**Keywords:** 21-hydroxylase deficiency, Congenital adrenal hyperplasia, Newborn screening, Public health

## Abstract

**Objective:**

This is a 10-year period, descriptive and retrospective evaluation of a Brazilian State NBS-CAH public program, bringing light to the prior discussion concerning its implementation, by sharing the screening-240,000 babies annually practices.

**Methods:**

The Minas Gerais (MG) NBS program has been coordinated by NUPAD, a neonatal screening, monitoring care, and genetics center at the Federal University of Minas Gerais (UFMG), intermediating all necessary actions, under the management of the State Health Administration, and following the Brazilian universal program. The dataset was used to calculate sensitivity, specificity, positive predictive value (PPV), incidence, number of carriers, and false-positive rates.

**Results:**

About 2,094,588 newborns were screened, and the incidence was 1:14,647; PPV was 10.5%. Most samples were collected on the fifth day after birth (interquartile range 4–6 days); 1352 babies were referred for clinical evaluation; 1210 were false positives (0.06%), and 142 presented the classic form; 22% of newborns were hospitalized due to salt-loss symptoms before or at the first visit.

**Conclusions:**

The MG NBS-CAH success as a public program comes from the Brazilian unified public health system, with integrated care, and from the newborn screening center offering timely referred screened positive cases and its outstanding state coverage. Appropriate cutoffs and close monitoring of hospitalized newborns have been pivotal for reducing the high false-positive rates.

## Introduction

Newborn bloodspot screening (NBS) programs for congenital adrenal hyperplasia (CAH) due to 21-hydroxylase deficiency (21OHD) have significantly reduced mortality in children with severe forms of the disease, through presymptomatic treatment shortly after birth, since the 1960s. Several recent reviews have attempted cost-benefit analysis of NBS CAH in countries that display good healthcare systems, policies, and facilities.^1^, ^2^, ^3^, ^4^

Despite the substantial benefits and high uptake of NBS CAH worldwide, healthcare providers still face a major set of challenges mainly due to high false-positive rates while screening for CAH.^5^, ^6^, ^7^ Several approaches have been tried to improve it, although false-positive results mainly from preterm and stressed newborns cannot be completely overcome while using 17-hydroxyprogesterone (17OHP) immunoassays.^8^

Liquid chromatography-tandem mass spectrometry (LC-MS/MS) is a core analytical technology in many clinical laboratories and has been advocated to improve NBS CAH accuracy, typically as a second tier test.^9^ Molecular testing has also been explored as a second-tier strategy to enhance the accuracy of CAH NBS.^10^ Both strategies imply additional costs to public programs and may negatively impact their feasibility, notably in larger middle-income stage countries, such as Brazil.^11^, ^12^

However, screening for CAH in developing countries might be cost-effective,^13^, ^14^ for the population and sustained by the public health system, but with other alternative strategies rather than LC-MS/MS second-tier testing to face high recall rates. Thereby, this paper sought to summarize the past ten years of experience of a large Brazilian program, the Minas Gerais State (MG) NBS CAH, aiming to bring light to the prior discussion concerning its implementation, by sharing the screening-240,000 babies annually practices.

## Methods

This is a descriptive and retrospective evaluation of the public NBS program for CAH in the state of Minas Gerais, Brazil, covering a 10-year period from 2013 to 2023 since its implementation. The study was approved by the Ethics Committee of the Minas Gerais Federal University (UFMG) - ETIC 392/07. The dataset was used to calculate sensitivity, specificity, positive predictive value (PPV), incidence, number of carriers, and false-positive rate.

Brazilian public law for NBS was amended in 2001 (Brazilian Public Health Law: Ordinance Ministry of Health No 822, 06/06/2001). In July 2012, screening for CAH was added to the national recommended panel (Ordinance Ministry of Health No 2829, 12/14/2012).^15^

The Minas Gerais NBS is coordinated by the State Health Department. The neonatal screening, monitoring care, and genetics reference center called *Núcleo de Ações e Pesquisa em Apoio Diagnóstico* (NUPAD),^16^ is located at UFMG. It intermediates all necessary NBS actions, together with the unified public health system network (*Sistema Único de Saúde*-SUS).

Blood spots on filter paper (S&S 903®) are collected at public primary care basic units (90%) or public birth hospitals (10%) between days 3 and 5, after birth, for 17OHP measurements. There are 3744 collection points. Dried blood specimens are mailed to the laboratory at the NUPAD reference center. A solid phase, 17OHP-time-resolved immunofluorometric assay is routinely used at NUPAD laboratory and determined by an integrated plate processor GSP® Neonatal 17α-OH-progesterone kit from Revvity.

The program currently employs a single-tier screening strategy with combined gestational age and birth weight-adjusted laboratory thresholds for 17OHP measurements. Term newborns (≥ 37 wk of gestation) are categorized by weight, as depicted in Table 1A. A specific protocol for preterm newborns (< 37 wk), also based on birth weight, has been recently introduced (Table 1B). Stressed newborns undergo serial testing based on their health status and are tested shortly before hospital discharge. A supportive team is responsible for clinical short-term monitoring. Presumptive positive results are directed to the healthcare provider in 24 h and NUPAD schedules the pediatric endocrinology appointment at UFMG outpatient care center (Hospital das Clínicas, Belo Horizonte), for confirmatory testing and follow-up.

**Table 1:** Cutoffs for 17-hydroxyprogesterone on filter paper for prior and repeated specimens, from the Minas Gerais State (Brazil) newborn bloodspot screening program for congenital adrenal hyperplasia (NBS CAH 2024).

A. Full-term birthweight categories	17OHP Cutoff^a^	
(≥ 37 wk)		
≥ 2500*g* (or not addressed)	15 ng/mL	(42 nmol/L)
2499–2000*g*	45 ng/mL	(136 nmol/L)
1999–1500g	89 ng/mL	(269 nmol/L)
≤ 1500g	190 ng/mL	(575 nmol/L)

B. Preterm birthweight categories	17OHP Cutoffs^a^	
(< 37 wk)		

≥ 2500	37 ng/mL	(112 nmol/L)
2499–2000*g*	59 ng/mL	(179 nmol/L)
1999–1500g	91 ng/mL	(275 nmol/L)
≤ 1500g	195 ng/mL	(590 nmol/L)

a Immunoassay, 17OHP-GSP® Genetic Screening Processor kit from Revvity.

**Figure 1:**
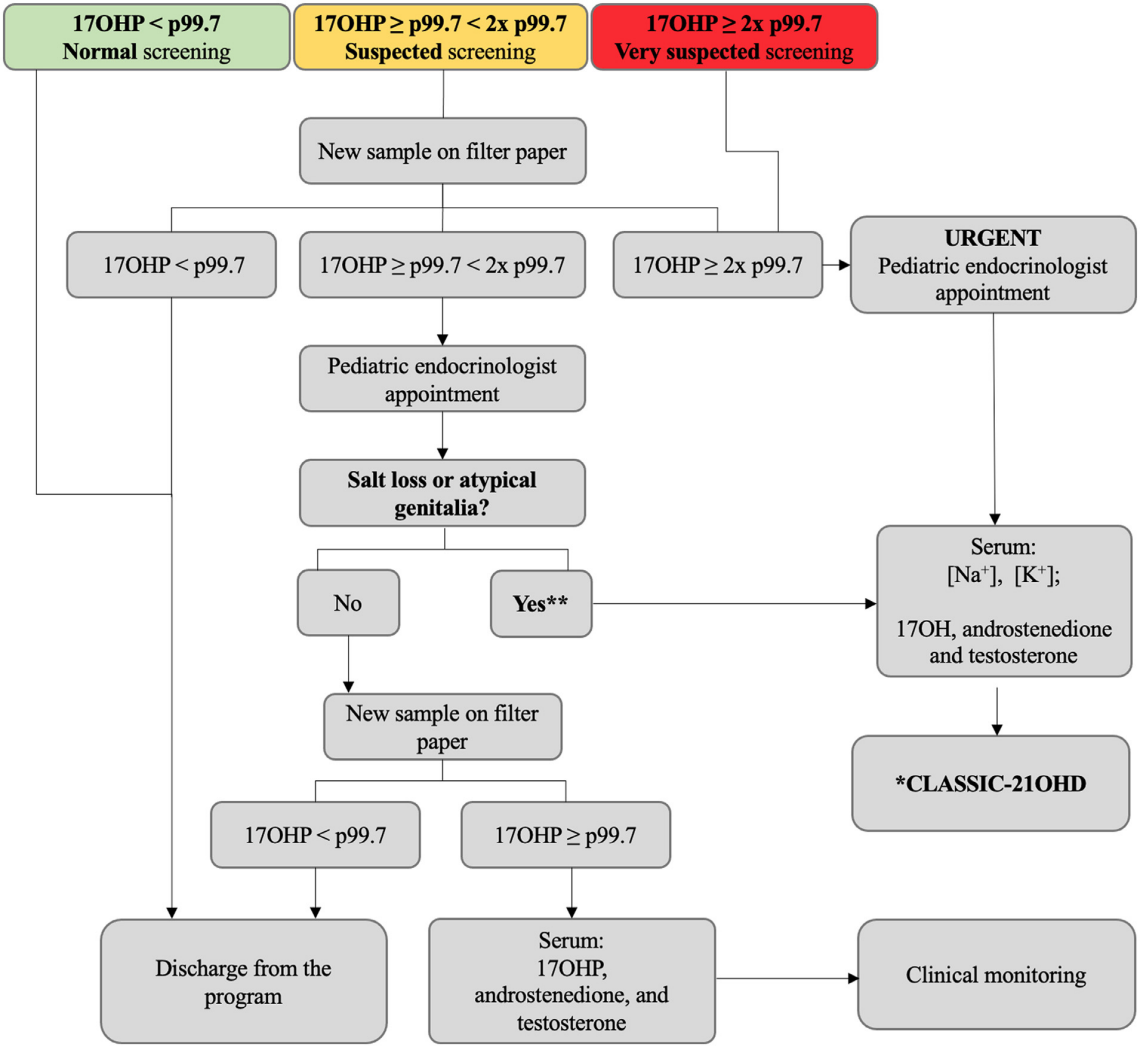
Current algorithm of the State of Minas Gerais (Brazil) newborn bloodspot screening program for congenital adrenal hyperplasia (NBS CAH 2024) for the diagnosis of the 21-hydroxylase classic form in newborns without neonatal complications (standard protocol). 17OHP, 17-hydroxyprogesterone; 21OHD, 21-hydroxylase deficiency; NBS CAH, newborn screening for congenital adrenal hyperplasia; p, percentile. *21-hydroxylase CAH-classic form: elevated 17OHP, androstenedione and testosterone (chemiluminescence). ^⁎⁎^Karyotype and ultrasonography for atypical genitalia investigation; psychosocial care and counseling.

The standard protocol is outlined in the algorithm below (Figure 1). Cutoffs for 17OHP blood spot measurements are set at the 99.7th percentile of 17OHP values from newborns in MG. These cutoffs are used for both first and repeated samples and are periodically reassessed. Newborns with 17OHP levels ≥ 2 times the 99.7th percentile are considered at higher risk for the disease and are promptly evaluated by the program. Confirmatory serum 17OHP, androstenedione, and testosterone are measured by chemiluminescence at the referenced laboratory, with reference values according to the child age.

Newborns undergo serial serum electrolyte evaluations right after the first medical appointment. The 21OHD classic form diagnosis is based on clinical and biochemical evaluation (elevated serum 17OHP, androstenedione, and testosterone), and salt-wasting (SW) subjects presented well-documented hyponatremia and hyperkalemia.

An integrative review of the subject was performed in Medline (PubMed), Lilacs (BVS), Scopus, and Web of Science databases with emphasis on more recent papers and in English; with the use of the terms: “newborn screening”; “newborn screening in Latin America”; “congenital adrenal hyperplasia”; “21-hydroxylase deficiency”; “screening for congenital adrenal hyperplasia”; “liquid chromatography with tandem mass spectrometry”; and “positive predictive value”.

## Results

### The program effectiveness

Minas Gerais is the fourth most extensive state (588,383 km^2^, larger than the metropolitan France) and the second in terms of population, located in Southeast Brazil. Its territory is subdivided into 853 municipalities, the largest number among Brazilian states.

The Minas Gerais public NBS is a state-run healthcare initiative, that primarily targets the SUS population of all municipalities. The program's coverage was 90%. From October 2013 to 2022, there were 2350,510 live births registered in Minas Gerais,^17^ and 2094,588 newborns were screened by the program.

Since 2013, samples have been collected on the fifth day after birth, and the interquartile ranged 4–6 days. Specimen collections are strongly recommended between 3 and 5 days. Late samplings (up to 30 days) are not encouraged by the NBS program, although rare cases have occurred.

The incidence of the 21OHD-classic form was calculated as 1:14,647; PPV was 10.5%. One girl with simple-virilizing (SV) form, whose NBS results were normal, was reported to the program and subsequently diagnosed as a false-negative case. Two other newborns were no longer located after the first sample was considered unsatisfactory. Sensitivity and specificity were around 99.3% and 99.9%, respectively.

### Confirmed cases

During the 10-year period of this study, 1352 babies were referred by the program for clinical evaluation. A hundred and forty-two newborns presented the 21OHD-classic form. The ratio of SW to SV was 2,6:1; 103 (73%) babies with SW (63 males/ 40 females) and 39 (27%) with the SV form (10 males/ 29 females). Six girls (8.7%), who were registered as males, had correct sex reassignment right after complete evaluation for both CAH and atypical genitalia conditions. Families received psychosocial care and counseling. Thirty-one newborns (22%) were admitted due to salt-loss symptoms either before or right after the specialist appointment.

Eighty-nine percent of CAH newborns were full term and 9% presented low-birth weight (< 2500 *g*). The low to normal birthweight proportion among SW subjects was 1:5, while no SV newborns were underweighted.

The median 17OHP concentration on the first dried blood spot of affected subjects was 243 ng/mL (735 nmol/L). Among SW subjects, the median value was 340 ng/mL (1025 nmol/L) and ranged 53.8–819; the median value was 51 ng/mL (154 nmol/L) and ranged 19.3–410 among SV subjects.

The clinical features of the confirmed cases are summarized in Table 2. All children with diagnosis have been followed up at the same reference institution (pediatric endocrinology outpatient care center at Hospital das Clínicas - UFMG), by a healthcare team that includes a psychologist, a geneticist, pediatric surgeons, and pediatric endocrinologists.

**Table 2:** Clinical features of confirmed cases from the Minas Gerais (MG) State, Brazil, newborn screening program (NBS CAH 2024) for congenital adrenal hyperplasia according to phenotype.

Age at first medical appointment (days old)		
Median	20	
Range	6–81	
Clinical Form	Salt-wasting form (*n* = 103)	Simple virilizing form (*n* = 39)
Sex		
Female	40	29
Male	63	10
Weight at birth		
< 2500g	17	0
≥ 2500g	86	39
Gestational age at birth		
< 37 wk	12	1
≥ 37 wk	78	33
not informed	13	5
First Neonatal 17OHP result (ng/mL)		
Median	340	51
Range	53.8–819	19.3–410
Hospitalization due to salt loss	22 (22 %)	

### False positives

Out of 2,094,588 screened newborns, 1210 (0.06%) had elevated initial 17OHP levels on dried blood spots and confirmatory serum tests, but the values subsequently normalized; therefore, these cases were false positives. Infants with false-positive results were discharged from the program soon after normalized serum 17OHP, androstenedione, and testosterone.

## Discussion

The incidence of classic-21OHD for the ten-year period, at Minas Gerais State was 1:14,647, which was higher than previously noticed (1:19,927).^18^ However, it surely represents the real statistics, considering the greater sample size. And it is, also, most like the reported by Brazilian databases^19^, ^20^, ^21^, ^22^ (1:10,000 to 1:15,000), as seen below, in Table 3.

**Table 3:** Brazilian newborn screening for congenital adrenal hyperplasia data from the regional program (NBS CAH 2024).^21^, ^22^, ^23^, ^24^

NBS Public State Programs in Brazil	CAH Incidence
Goiás	1:10,000
São Paulo	1:10,460
Rio Grande do Sul	1:13,551
Minas Gerais	1:14,647^a^
Santa Catarina	1:14,972

a Current data. CAH, congenital adrenal hyperplasia; NBS, newborn screening.

NBS was incorporated into the public and unified system network (SUS) at MG State in 1993, testing for both phenylketonuria and congenital hypothyroidism conditions. Other federative units also began to organize and include the NBS practices into their public regional networks, but it was only in 2001 it was created a Brazilian universal program with a focus on early intervention and permanent monitoring.^23^

The actions are articulated between the Federal Ministry of Health, the 26 states’ health departments (in addition to the Federal District), and its 5570 respective municipalities. The implementation of the Brazilian national program has been gradual primarily due to regional inequalities in health facilities. The first four diseases (congenital hypothyroidism, phenylketonuria, sickle cell disease, and cystic fibrosis) were proposed to be included in two steps.

In 2013, screening for CAH and biotinidase deficiency was added to the initial list of diseases as part of the state programs, including Minas Gerais. An extensive panel of diseases (ex.: congenital toxoplasmosis, organic acid conditions, fatty acid oxidation disorders, amino acid disorders, lysosomal diseases, immunodeficiencies, and spinal muscular atrophy) was recently approved by the Brazilian Ministry of Health and has been progressively implemented. Brazilian NBS programs have been improved but with large regional variations, both in magnitude and in coverage trends over the past ten years. A recent report on newborn screening testing shows the existence of inequalities of access according to the region of residence, income, and health insurance, and highlights the need to develop strategies to promote universal access and equity.^24^

NBS for CAH has been challenging for all national or regional Latin American programs’ providers largely due to the often-high screening false-positive rates. Immunological assays often used for NBS screening overestimate the 17OHP levels due to the low specificity of antibodies, and the cross-reaction that occurs with hormones produced by the immature adrenal. This issue leads to low PPV for the first-tier screening (estimated to be <10%), as seen in the present series for the first pilot period.^18^ Thus, a key challenge faced while screening for CAH has been to determine the cutoff value for 17OHP, that will result in adequate cost-benefit.

Cuba, Costa Rica, and Uruguay were pioneers in implementing national NBS CAH programs in Latin America. Brazil has implemented in the past decade as Argentina, which shows the highest incidence (1:8937), Mexico, and Panama. Other countries have recently started their programs, such as Ecuador, Peru, and Bolivia. Several combined approaches, other than second-tier LC-MS/MS, have been developed in Latin America to improve screening outcomes: firstly, organic extraction to remove cross-reacting steroid sulfates; secondly, adjusting cutoff levels to birthweight or gestational age, and age at sample collection.^25^ Uruguay and Costa Rica have the most comprehensive public programs in Latin America, having implemented expanded newborn screening using MS/MS at a national level.^26^

The Minas Gerais NBS-CAH program has become more effective since its implementation, with the positive predictive value (PPV) increasing from 2.1%^18^ to 10.5%. The authors have overcome the high false-positive rate observed during the previous pilot phase through improved assays (removing cross-reacting steroid compounds) and appropriate cutoffs. The program has been highly successful in providing diagnosis, treatment, follow-up, and education to parents and providers. These advancements are due primarily to the public unified healthcare system with integrated patient care and monitoring. Additionally, the success can be attributed to the coordinated efforts of the NBS program center, NUPAD, which ensures timely referrals; continuous monitoring of cases in birth hospitals; outstanding state coverage; and long-term follow-up of NBS patients with regular appointments at the reference hospital.

Although there has been good coverage by the NBS public program, recent increase in the availability of tests with expanded panels in the private healthcare system using dried blood spots collected during birth hospitalization for individuals insured by health plans has been observed. Screening in the private health system does not meet the notification and active search requirements, which may hinder good prognosis for children affected by the disease. The absence of integration in NBS follow-up between the public and private sectors coupled with the complete lack of official information on NBS in the private sector hamper an accurate assessment. In Brazil, the coverage of the Guthrie test increased from 96.5% to 97.8% of newborns tested by both public and private sectors together from 2013 to 2019. Nonetheless, there are significant inequalities in the performance of screening tests, with higher rates among children whose families reported higher per capita household income, those living in the South and Southeast regions, and those with private health insurance.^27^

The CAH-program multidisciplinary team also supports families of virilized girls and promotes correct sex reassignment. Although not a goal of newborn screening, some children with the non-classic form (NC—CAH) have been diagnosed in this series.^28^

Although gestational age is a better parameter, the Minas Gerais NBS has stated for ten years only birthweight categories for 17OHP cutoffs because gestational age was neither widely available nor reliable in all state regions. Recent improvements in data management have enabled gestational age to be currently used, resulting in a significant 40% reduction in referrals for new appointments, over the past 9 months.

Adjustments to cutoffs depending on age at sampling were not considered for the Minas Gerais population, because only a few samples were performed < 48 h, a critical period for elevated 17OHP values. Perinatal complications (pregnancy-induced hypertension, early onset sepsis, neonatal seizures, and birth asphyxia) brought additional concerns for the interpretation of screening results. The present program's experience showed that close monitoring and follow-up testing of hospitalized newborns have been an essential strategy to improve the CAH screening precision and reduce recall rates.

Preterm births are the main reason for false-positive CAH screening tests, as the authors reported before.^18^ Even among healthy border preterm infants, 17OHP concentrations are usually higher due to immature adrenal function, which leads to an increase in the concentration of precursors in relation to the final metabolites of adrenal steroidogenesis. Most Latin American countries are near the average of 9.5% of births being preterm. Colombia is the only one significantly above average with nearly 15% of preterm births, followed by Brazil with 11%.^12^ So, both countries may present additional challenges while testing not-hospitalized preterm newborns for CAH. Establishing specific cutoffs for these babies and conducting further reassessments have proven to be effective strategies for reducing recall rates and are currently being implemented in this program.

Early CYP21A2 genotyping has been a valuable complement to the 17OHP analysis to predict disease severity, make treatment decisions and for the follow-up and evaluation of screening programs where there is high incidence of the disease,^29^ although the timeline to complete the molecular protocol might not be suitable for some countries or regions.

Further studies based on long-term outcomes should be performed to better address it. In the studied cohort, the authors found a high frequency of NC—CAH diagnosis in children with persistent elevated 17OHP levels, supporting molecular study as decisive for elucidating these cases.

The 21-deoxycortisol (21-deoxy) is not elevated in healthy stressed infants, and this metabolite would fit as a better biomarker than 17OHP for CAH diagnosis, but 21-deoxy assays have not been available yet.^30^ Novel steroid profile assessments would also improve screening bring advantages to the studied population, and further decrease follow-up time and the number of false-positive referrals.

Certainly, several challenges must be addressed to achieve the goal of treating most children with CAH as early as possible. It is important to acknowledge the main challenges faced in maintaining the Minas Gerais NBS-CAH program. Firstly, Minas Gerais is a large state, and despite the remarkable work of the monitoring and control sector at NUPAD, its size may contribute to delays in initiating treatment. As a result, presymptomatic diagnosis for SW subjects may be compromised due to logistical issues in transporting samples and even patients, as evidenced by infants who were hospitalized before their first appointment.

According to the Working Group on Neonatal Screening of the European Society for Pediatric Endocrinology,^31^ the results of NBS CAH should ideally be available within ten days after birth. To date, although the time to collect the first spot sample is in the expected range, eighty-five percent of SW subjects were diagnosed after 2 wk of life, causing treatment delay, but with no deaths.

Another significant challenge is that medical care for newborns is centralized at a single pediatric endocrinology center, where patients with abnormal blood spot results have regular appointments with specialists from the NBS-CAH program. Given the large size of the state, this situation poses a problem for families living far from the NUPAD reference center. Nevertheless, enhancing care in remote regions, including access to specialists, is essential to address this issue.

Children with the classical form, who were hospitalized before their scheduled medical appointments, or during their first visit, came from remote regions of the state. To address this issue, newborns with elevated 17OHP in dried blood spots are monitored, and the local medical team is promptly guided by the pediatric endocrinologists of the program by phone contact at NUPAD. Likewise, the first CAH screening results have guided the clinical reasoning for the atypical genitalia investigation among girls who were not discharged after birth.

Moreover, there was a significant expansion of telehealth use for NBS CAH false-positive referrals at the onset of the COVID-19 pandemic.^32^ Recently, because of this experience, the authors have successfully managed to reduce false-positive appointments. So, telemedicine might be a viable alternative to in-person services in large states, such as Minas Gerais, in the future.

The authors have overcome the high false-positive rate seen in the previous pilot period^18^ using improved assays, establishing new appropriate cutoffs, and close monitoring of hospitalized newborns. Data from larger cohorts revealed that the sensitivity of the CAH NBS is strongly correlated with the duration of follow-up and that the recall rate in full-term infants is lower than that in preterm infants.^33^

In Brazil, despite the NBS CAH has been mandated since 2013, its implementation is far from expected, with significant inequalities across the country. In 2020, coverage reached 82,5% and the median age for the first medical appointment for infants with abnormal CAH screening tests was 30 days.^34^ Data indicates that screening programs in Latin America have experienced significant growth over the years, but impaired access to health care remains a challenge.

## Conclusions

The present data highlights the critical role of NBS in reducing mortality and morbidity associated with CAH, even amidst challenges related to assisting large populations within a public healthcare system. The CAH program in Minas Gerais will continue to deal with new challenges, particularly the need for greater involvement of public health authorities in the assistance network. A significant priority is to enhance communication with the private sector, which has been increasing the number of expanded newborn screening tests but lacks an effective follow-up system. So, close collaboration between local public primary care providers and private pediatric services will be essential in the future.

The first 10-year experience after screening two million newborns for CAH shows that:

• Programs with single-tier screening can achieve an outstanding outcome but require a major organizational resource for short-term follow-up.
• A multidisciplinary care network is essential for the success of programs, particularly in the treatment of children with intrauterine virilization.
• Appropriate cutoffs and close monitoring of hospitalized and premature infants yield to acceptable false-positive rates, without using LC-MS/MS as second-tier testing.
• Collecting specimens at 4–7 days in primary care clinics and conducting follow-up of positive screening infants both eliminate one of the major barriers to NBS in public health NBS programs, which is the loss of follow-up after screening.
• Serious morbidity and mortality can be prevented from late CAH diagnosis, even if results are reported after 14 days of life.
• Active search and long-term follow-up of positive screening infants coordinated by the NBS program is an essential tool for successful interdisciplinary care.

## Funding

This research received no external funding.

## Author contributions

Conceptualization, methodology C.B.B and I.N.S; investigation, C.B.B, G.S.S, T.R.V, R.F.A, H.P.O, A.L.A.C.P.G and R.M.M; formal analysis, C.B.B and G.S.S; original draft preparation, C.B.B; writing, review and editing, I.N.S, and J.N.J; supervision, I.N.S. All authors have read and agreed to the published version of the manuscript.

## Conflicts of interest

The authors declare no conflicts of interest.
